# The Descriptive and Physiological Anatomy of the Brain, Spinal Cord, and Ganglions, and of Their Coverings. Adapted to the Use of Students

**Published:** 1846-04

**Authors:** 


### ART. X.

*The Descriptive and Physiological Anatomy of the Brain, Spinal Cord, and Ganglions, and of their Coverings. Adapted to the Use of Students.* By ROBERT BENTLEY TODD, M.D., F.R.S., Professor of Physiology in King's College, London, &c.—*London,* 1845. Small 8vo, pp. 284. With 38 wood-engravings.

THIS little work is a reprint of the article "Nervous centres," in the 'Cyclopædia of Anatomy and Physiology,' with several additions, and some alterations, suggested by its reperusal as it again passed through the press. It owes its appearance, as a separate volume, to the frequent complaints which have reached the author, from students and others, of the want of a description of the Brain and Spinal Cord, embodying the most recent observations on the anatomy of these important organs. These complaints we regard as well-founded, but we are not quite sure that the present reprint is the means best fitted to allay them. We are confident that there is no part of the human body, which so fully needs all the illustration and aid to be derived from comparative anatomy, as the nervous system requires; and we are sorry, therefore, that any treatise, expressly devoted to it, should exclude the valuable information afforded by the researches, that have been recently made upon other types of structure. As an exposition of the structure of the nervous centres in Man, however, we are disposed to regard it as the most full and accurate that has yet appeared in this country, and to recommend it to the attention of the student accordingly.

Prefixed to the anatomical details, which constitute the bulk of the volume, is a series of "Aphorisms respecting the nervous system," which presents, in a very clear and concise form, the chief truths which have been ascertained in regard to its functions. We must take exception, however, to the language in which some of them are couched, as tending to mislead the reader; though we cannot believe that Dr. Todd's ideas on the subject are really different from our own. "The nervous force," he justly observes, "is a polar force, resembling electricity in the instantaneousness of its development, and in the rapidity of its propagation, but differing from it in several important features. It is a peculiar feature of the nervous force, not only that it may be developed under the influence either of a mental or of a physical stimulus, but that, being excited by a physical stimulus, it is capable of affecting the mind." So far, so good. The nervous force itself is recognized as the same, whether excited by the will acting through the brain, or by an irritation applied to the trunk of the nerve; and this is in accordance with sound philosophy, which forbids us to regard as different the conditions into which the nerve is thrown by these two agencies,—the sole manifestations of those conditions, exhibited through the muscles, being precisely the same. But, continues Dr. Todd:

"14. Hence nervous actions may be conveniently distinguished into two classes, according as the mind participates in them or not; namely, mental and physical.

"15. Mental nervous actions are those which originate in, and are excited by, an act of the mind, as all voluntary actions; or which, originating in a physical impression, produce an affection of the mind, as in all sensations.

"16. Physical nervous actions are those which take place without the *necessary* intervention of the mind, and which result from a physical exciting cause. Of this kind are all those actions which are caused by a physical change, frequently morbid, in a centre or in a nerve, and those which result from the excitation of the vesicular matter in which a motor nerve is implanted, by the stimulation of the peripheral fibres of a contiguous sensitive nerve. These latter have been designated *reflex actions;* and although most of them, in health, are attended with consciousness, still this mental state is in no way necessary to the perfection of the nervous act, and should be regarded rather as an incidental accompaniment to it, than as an essential part of it." (p. xii.)

Now we cannot but regard the terms "mental nervous actions," and "physical nervous actions," as utterly meaningless, or as involving ideas altogether incongruous. If it be admitted that the "polar" condition of the nervous system itself is the same, whether it be excited by a mental or a physical cause, where is the distinction between the actions which are the manifestation of that condition? Should we say of the fall of a stone to the earth, that it was a case of "mental gravitation," if it were let go by an act of human will, but of "physical gravitation," if it were thrown down by the wind, or detached by a stroke of lightning? The idea that the "polar" condition of the nervous system is itself different,—according as, in the one class of changes, it is destined to produce sensations or to excite reflex actions,—or as, in the other class, it originates in the will or is produced by a physical cause,—appears to us quite unsupported by evidence; and we are satisfied, from the previous aphorisms, that Dr. Todd has no intention of propounding it. What, then, can be the meaning of the terms "mental nervous," and "physical nervous?" The *nervous* action is one thing, the *mental* action another. The nervous action is always physical; that is, it is comparable in its nature (so far as this is known to us), and in the conditions on which this is dependent, with other physical actions. Of the nature of mental action we know nothing, it is completely unlike everything else, "none but *itself* can be *its* parallel." But we know that it is connected, in some way that we cannot comprehend, with the proper physical actions of the nervous system; being stimulated by the latter, when the part that is in action is the one that has the power of exciting sensations in the mind; and in its turn being the stimulus of the latter, when the will, or an emotional or instinctive impulse, is directed to the production of muscular movement. Thus, then, nervous action, itself physical, may be produced or stimulated by *physical* causes, or by *mental* causes; and it may be productive of *physical* or of *mental* changes, according to the part of the nervous system concerned. But such a difference in the *causes* or in the *effects* of the action does not warrant the combination of terms employed by Dr. Todd; the only excuse for which would be looked for in a difference in the action itself, which is not admitted by Dr. Todd any more than by ourselves.

We shall make a few quotations from the anatomical portion of the work, which may give an idea of the clearness and amplitude of its descriptions. After entering at some length into an account of the cranio-spinal fluid, to which particular attention has been drawn by Magendie, Dr. Todd thus continues:

"Practical men are too much in the habit of attributing morbid phenomena of the nervous system to the influence of the pressure of a liquid effusion upon the brain or spinal cord. Many facts tend to show, that, in a large proportion of cases, especially in the adult, the occurrence of an increased quantity of fluid, either around those centres or within the ventricles, is *a result*, and that it is probably a result *of a conservative kind*, consequent upon a morbid change which depresses the general nutrition of those organs themselves. We have seen how the universal decay of the tissues, which characterizes old age, favours the increase of the cranio-spinal liquid, when it affects the brain and spinal cord. In examining the bodies of habitual drunkards, patients who die of delirium tremens, or of cirrhosis of the liver, the quantity of fluid is always found to be considerable, and the brain shrunk. In bed-ridden persons, who have ceased to exercise their faculties for some time, whether for mental or bodily exertion, the same phenomena are witnessed. When there has been much anæmia, as in cases where death has terminated a protracted illness, in phthisis for example, or in persons who have died of hemorrhage, or after excessive venesection, the nervous centres will be found to be small, and the liquid in large quantity. In extreme cases of lead cachexy, in which the nutrition of the nervous and muscular tissues is materially diminished, I have observed similar appearances. And, when any partial atrophy of either the brain or the spinal cord has occurred, there will invariably be found, at a point corresponding to it on the exterior of the organ, a local accumulation of fluid, occupying a depression on its surface, which has been caused by the giving way of the nervous substance within.

"On the other hand, an increase in the quantity of the nervous substance, or an enlargement of the brain or spinal cord, consequent on an undue injection of their blood-vessels, is invariably accompanied with a diminution in the quantity of this fluid, or with the total absence of it. In hypertrophy of the brain, no fluid is found in the subarachnoid space, and very little or none in the ventricles. In cases of tumour of the brain encroaching upon the cranial cavity, we find no fluid; and the same is observed where chronic inflammation of the brain has given rise to a new deposit, which increases the bulk and the density of the cranial contents. In all cases where a considerable quantity of fluid has accumulated *within* the ventricles, that upon the surface is either greatly diminished or entirely disappeared. In the ordinary hydrocephalus internus of children, fluid is never found on the exterior of the brain.

"When an arrest in the development of any portion of the cerebro-spinal axis has taken place, the space which ought to be occupied by the organ of imperfect growth is filled by liquid. In examining the heads of idiots, we always find a considerable quantity of sub-arachnoid fluid, either general or partial, if a portion only of the brain be deficient." (p. 50.)

We fully agree with Dr. Todd in his general deduction, that the preternatural increase of this fluid should be usually regarded as secondary to the diminished size of the cerebro-spinal centre itself, or to the diminished quantity of blood in the vessels, and that it has little or no direct connexion with the manifestation of peculiar symptoms during life, in the great majority of instances. The complete investment of the encephalon by an unyielding bony case, obviously places it in a different condition from any other organ, since any diminution in its bulk must leave a vacuum (which Nature, *here* at least, seems to abhor), if the space be not filled up with fluid. But it would be absurd to deny that the cerebro-spinal fluid occasionally undergoes a preternatural increase, whilst the brain experiences no diminution in bulk, or itself becomes enlarged. This increase, however, rarely takes place in the sub-arachnoid fluid of the exterior of the brain, being much more common in the fluid within the lateral ventricles; the walls of which acquire, after they have been long subjected to its pressure, a preternatural degree of firmness, so as not to collapse when laid open. In these views Dr. Todd accords with the opinions first brought forward by the late Dr. Sims, in his valuable paper on serous effusion in the brain.

Dr. Todd has minutely examined the structure of the Pacchionian bodies, and considers them as morbid structures originating in a deposit of lymph among the vessels of the pia mater, which pushes the arachnoid before it as an investing sac. In some instances the granular mass is only partially covered, and then it causes only a slight projection on the surface of the visceral layer of arachnoid; but in others the mass is completely covered, and a stalk is gradually formed; and when several granular masses have been deposited immediately contiguous to each other, they may all be attached by a cluster to the same stem. The membranous sacs of some of the bodies have epithelial particles upon their surface, whereby the derivation of these sacs from the arachnoid membrane is made apparent.

"The great frequency with which these bodies are met with in the various situations above mentioned, has induced many, even in the present day, to regard them as normal structures, the physiological office of which is as yet unknown. But there are many facts which strongly militate against such a conclusion. In the first place it may be observed, that the Pacchionian bodies never occur in the earliest periods of life. In the course of a long experience in anatomical investigations, I have never seen them at a period antecedent to six years. The brothers Wenzel, who made a series of special examinations, with a view to determine this question, make the following statement:—In children, from birth to the third year, these bodies, if they ever occur, must be very few. From the seventh to the twentieth year, they sometimes are numerous. From the latter period to the fortieth year, their number is considerable, and the nearer we approach the fortieth year, the greater does it become. Lastly, from the fortieth to the one hundredth year, these bodies are found in great numbers.

"It must further be remarked, that even at those periods of life when the Pacchionian bodies are found in greatest numbers, cases frequently occur in which no trace of them can be found. There is likewise the greatest variety, as to their number and size, in different individuals of the same age. It has always occurred to me to find them most numerous in cases, where I had reason to know that the brain had been subject to frequent excitement during life. In persons addicted to the excessive use of spirituous liquors, in those of irritable temperament, and who had frequently been a prey to violent and exciting passions, they are almost uniformly highly developed. The Pacchionian bodies are peculiar to the human subject, nothing similar to them has been found in any of the inferior classes of animals.

"In reference, then, to the question, what is the nature of these bodies, I have no difficulty in stating my opinion that the evidence greatly preponderates in favour of their morbid origin: that they are the product of a chronic, very gradual, irritation, due to more or less frequent functional excitement of the brain itself. It is not unlikely that the friction to which the opposed surfaces of the arachnoid are continually subjected in the movements of the brain, especially when they are of a more rapid and violent kind, as under states of cerebral excitement, may contribute to the development of many of the appearances connected with these bodies. The opaque spots, which are of such frequent occurrence upon the surface of the heart, may be quoted as an example of a morbid change, very commonly met with, and resulting probably from the friction against each other of opposed serous surfaces. Were the Pacchionian bodies normal structures, they would not be so frequently absent from brains which afforded every other indication of being in a healthy state; nor should we find opacity of the arachnoid (a decidedly unhealthy condition) so commonly coexistent with the full development of them. Again, were they a necessary part of the healthy organism, we might expect to find them more constant as regards size, number, and the extent of surface over which they were placed." (p. 60.)

This last argument is, perhaps, the weakest; since we *do* find an almost equal variation in the number and situation of the curious *Pacinian* corpuscles; which can hardly be regarded in the light of morbid structures.

The description of the vesicular nervous matter, which is peculiarly full and lucid, closes with the following well-expressed queries:

"The great simplicity in the form of the elements of the gray nervous matter is one of its most remarkable characteristics. That a tissue, which plays the prominent part in the nervous actions, whether they are prompted by mental change or are purely corporeal, should exhibit scarcely any more complexity of structure than that which is found in the simplest animal or vegetable textures, or in structures that have not passed their earliest phase of development, is an anatomical fact pregnant with great physiological interest. Have this simplicity of form and delicacy of structure reference to the celerity of the nervous actions ? or to that proneness to change which must be induced by the constant and unceasing round of impressions, which the gray matter must receive from the ordinary nutrient actions that are going on in the body, as well as from the continual action of thoughts? If, according to common acceptation, we admit that the mind has some immediate connexion with the cerebral convolutions, it may well be imagined that no part of the frame can be the seat of such active change, from its being, on the one hand, the recipient of impressions from the body, and, on the other, from an association with the psychical principle so intimate that probably, under ordinary circumstances, an affection of the one cannot occur without being communicated to, and producing a change in the other.

"Another curious fact, in connexion with the intimate structure of the gray nervous matter, is the large quantity of pigment or colouring matter which exists in it, and which appears to form one of its essential constituents, more abundant in some situations than in others, but present in all. We are utterly ignorant of the design of this peculiarity of structure. If this pigment bear any resemblance in chemical composition to the colouring matter of the blood, *hæmatosine,*—and it is not improbable that it does,—an increased interest attaches to the practical importance of minute attention, on the part of practitioners, to avail themselves of all the means which are capable of improving that important element of the nutrient fluid both in quantity and quality ; for it is most reasonable to presume that the pigment of the nervous matter would derive its nutriment from that of the blood." (p. 72.)

In regard to the connexion between the nutrition and waste of the vesicular matter of the nervous system, on the one hand, and the degree of its vital activity, on the other, our own mind has long been completely made up ; and we have, on several former occasions, expressed our conviction, with some of the reasons on which it is founded, in the pages of this Journal. The idea started by Dr. Todd, in regard to the relation between the pigment-granules, which seem to form an essential constituent of the cells of the gray matter, and the hæmatosine, which is contained within the red corpuscles, and which is manufactured (so to speak) by them, appears to us to be one of peculiar interest; and it derives increased weight from the disturbance of the functions of the nervous system, which invariably manifests itself, when there is any considerable deficiency in the proportion of red corpuscles in the blood.

By way of sample of the descriptions of the structure of the brain itself we may quote the following passages relative to the Mesocephale ; a term which may be new to some of our readers, but which was first suggested by Chaussier, as applicable to that part of the encephalic mass, in which the fibres of the different parts come into close relation.

"The pyramidal and olivary columns may be readily traced, as already explained, from the medulla oblongata up to the cerebral hemispheres, the former becoming united chiefly with the corpora striata, the latter with the optic thalami. In that part of their course which is intermediate to the medulla oblongata, these columns become mingled with certain transverse fibres, and with more or less of vesicular matter, and with them contribute to form a mass which is the connecting link between all the segments of the cerebellum, and may be compared to a railroad station, at which several lines meet and cross each other." (p. 180.)

We think that this simile may be carried a little further, since the mesocephale is not only a point for the intercommunication of different tracks, but is also a centre from which *new* trains may start, in virtue of the vesicular matter which forms a part of it. We are constrained to notice that the preceding extract does not afford a very favorable sample of Dr. Todd's style. We are at a loss to attach any distinct idea to the words, "intermediate to the medulla oblongata;" we know that one thing may be intermediate between *two* others, but to say that it can be intermediate to *one* other, is surely rather Irish than English. Again, we cannot but think that Dr. Todd meant to say that the mesocephalic mass is the connecting link between the segments, not of the cerebellum alone, but of the encephalon generally. We have noticed more than one important error of this description, which is obvious enough to the well-informed reader, but which is calculated to mislead the student. For example, in the description of the corpus callosum (p. 234-5), we find that, by dissection in a hardened brain, "it may be detached from the substance of the hemispheres as far outwards as the external border of the corpus *callosum* and optic thalamus." It is evidently the corpus *striatum* which is referred to in the last clause.—To return, however, to the mesocephale. After a full description of the several parts that enter into its formation, Dr. Todd continues:

"From the preceding description of the mesocephale, it may be concluded that two classes of elements enter into its formation. These are *intrinsic* and *extrinsic*. The former consists of the masses of vesicular matter, with which the fibrous matter, whatever be its course, is intimately connected. Such are the gray matter of the quadrigeminal bodies, that light gray matter which surrounds the olivary columns in their upward course, the darker matter which intervenes between the transverse fibrous lamellæ, and, more in front, that which forms the locus niger of the crus cerebri.

"The extrinsic elements are those which pass through this segment, being continuous with some portion of a neighbouring segment, or serving to connect the gray matter of the mesocephale with the hemispheres of the cerebrum or cerebellum, or with the medulla oblongata. The fibres which form the inferior layer of the pons are perhaps the only element that does not connect itself in some way with the gray matter of the mesocephale, since they seem simply to pass across from one crus cerebelli to the other. The deeper transverse fibres, the pyramids, the olivary columns, the processus cerebelli ad testes, all connect the neighbouring parts with the intrinsic matter of the mesocephale.

"It is plain, then, that anatomy affords abundant grounds for the conclusion that the mesocephale must be regarded as a distinct centre, connected by numerous bonds of union to the other segments of the brain." (p. 187.)

Correct as this description is in regard to the human brain, when considered independently, we still think it highly objectionable to include the corpora quadrigemina as a part of the mesocephale; since we learn from comparative anatomy that their analogues in the lower animals are as distinct from all the other divisions of the encephalon, as these are from each other. In regard to the Olfactory lobes, Dr. Todd fully develops the correct view, and justly remarks that it is high time for the designation of olfactory *nerves* to cease, as applied to those processes of cerebral matter, which lie upon the upper surface of the cribriform plate of the ethmoid bone. He points out their distinctly ganglionic character, as evinced by the intermixture of vesicular with fibrous structure; and he notices the ventricle contained in the olfactory bulb, which is evident enough in the lower mammals, as well as in the human fœtus at an early period. Why, then, if it be desirable to impress on the student correct notions regarding the nature of this body, does he include it among the encephalic nerves? Surely, in a scientific description of the brain, it ought to occupy as conspicuous a place as the mesocephale and the optic ganglia. And, we would add, is not the gray matter of the olivary columns really to be considered as forming auditory ganglia, connected as those columns are with the implantation of the auditory nerve?

We have looked with especial interest at those portions of the Treatise which relate to connexion between the spinal cord and its nerves; a question of particular importance, in regard to the physiological inferences to be derived from anatomical research, and one upon which there is still much discrepancy of opinion. Many of our readers doubtless recollect the time, when the spinal cord was commonly taught to be little else than a bundle of nerves proceeding from the brain, and when it was supposed that *all* the fibres which enter it from the nerve-roots pass upwards to the latter organ. This always seemed to us to be Sir C. Bell's view of its nature. When Mr. Grainger first enunciated the fact, that *some* of the fibres of each root lose themselves in the gray matter of the cord, his statement was received with suspicion. Yet now this seems abundantly confirmed; and with some anatomists the tide of opinion sets just the other way, their question being, whether *all* the fibres of the roots of the spinal nerves are not thus lost in its gray matter, and whether *any* have a direct connexion with the brain. Dr. Todd states that he is satisfied, by his own dissections, of Mr. Grainger's accuracy, in regard to the passage of certain fibres of the roots of the nerves into the *gray* matter of the cord; but he seems very doubtful as to the possibility of tracing the continuity of any of the nerve-roots with the *white* strands, as represented by Mr. Grainger, and consequently as to the existence of a distinct set of cerebral fibres. Of the former he says:

> "From a careful review of the preceding statements, it is plain that a large number of fibres pass into the gray matter of the cord, and probably form some intimate connexion with its minute elements; and this fact is favorable to the supposition that the spinal nerves derive their origin, at least partly, from the gray matter. It must be admitted either that these fibres unite in some way with the vesicles of the spinal gray matter, or that they pass through it up to the brain, in the gray matter of which they become implanted; the former seems the most reasonable supposition, and more consistent with the apparent oblique or transverse direction which the fibres take in the gray matter." (p. 273)

We may then regard the title of the spinal cord to the character of a distinct ganglionic centre, to be anatomically as well as physiologically proved. For a solution of the difficulty under which Dr. Todd labours, in regard to the connexion between the roots of the spinal nerves and the brain, through the white or fibrous portion of the cord, we would again refer him and other anatomists who experience the same difficulty, to the ventral cord of the articulata, which differs in no other obvious particular from the spinal cord of vertebrata, than in the want of continuity of the ganglionic portion, so that a number of distinct centres are formed by it, instead of one long tract; and this is in strict accordance with the general character of the group, which is that of segmental independence. We cannot, from our own investigations, feel the least doubt that a portion of the roots of the nerves of those animals is directly continuous, through the fibrous tract, with the cephalic ganglia, which represent the brain of vertebrata, whilst another portion has a distinct connexion with the ganglia of the cord. The fact is the more valuable since it was first announced by Mr. Newport, without any reference to the physiological inferences which are *now* founded upon it.

We now take our leave of this Treatise; cordially recommending it to the student as being, in spite of its defects, by far the best guide to the study of the Anatomy of the Human Brain which we possess. When it arrives at another edition, we would suggest to Dr. Todd whether it might not be advantageously increased to a small extent, by the introduction of a little more comparative anatomy,—not in a systematic form, but in such a shape as to illustrate some of the obscurities, which are inseparable from the isolated study of the human brain, and which are still numerous enough, when all the light has been thrown upon it that can be collected from extraneous sources. We may hint, also, that the wood engravings, some of them very elaborate ones, which add much to the value of the descriptions, would be rendered more useful, as well as more pleasing to the eye, by a higher style of presswork. It is lamentable to see so many good drawings, which have all the appearance of being carefully engraved, treated with such injustice by the printer.

---

